# Evaluation Approach of Fracture Behavior for Asphalt Concrete with Different Aggregate Gradations and Testing Temperatures Using Acoustic Emission Monitoring

**DOI:** 10.3390/ma14164390

**Published:** 2021-08-05

**Authors:** Liuxu Fu, Yubo Jiao, Xianhua Chen, Mengsu Zhang

**Affiliations:** 1 School of Transportation, Southeast University, Nanjing 211189, China; fulx@seu.edu.cn; 2 Key Laboratory of Urban Security and Disaster Engineering of Ministry of Education, Beijing University of Technology, Beijing 100124, China; jiaoyb@bjut.edu.cn; 3 School of Transportation, Jilin University, Changchun 130025, China; zhangms@jlu.edu.cn

**Keywords:** asphalt concrete, fracture behavior, aggregate gradation, temperature effect, acoustic emission

## Abstract

Different aggregate gradations of asphalt concrete possess dissimilar skeleton structures, leading to diverse macroscopic and mechanical characteristics. Acoustic emission (AE) technology can realize real-time monitoring of the whole damage evolution process of materials. The objective of the present investigation was to demonstrate the fracture characteristics of asphalt concrete with three types of aggregate gradations, including dense-graded asphalt concrete (AC), stone mastic asphalt (SMA), and open-graded friction course (OGFC) under indirect tensile load on account of the acoustic emission (AE) technique. The Marshall compaction method was used to prepare specimens, and the indirect tensile test (IDT) and AE monitoring were conducted simultaneously at different temperatures. The corresponding AE parameters containing energy, cumulative energy, count, and cumulative count were adopted to characterize the fracture process of asphalt concrete with different aggregate gradations. The impact of temperature on the damage characteristics of asphalt concrete was also assessed. Test results indicated that the AE parameters could effectively classify the damage stages of asphalt concrete, and specimens with different aggregate gradations exhibited different AE characteristics during failure processes. The combination of AE parameters and cumulative AE parameters can accurately characterize the damage characteristics of asphalt concrete. SMA specimens possessed the best overall performance among these three types of asphalt concrete in terms of the variations in energy and cumulative energy at different temperatures. The findings obtained in this study can provide a practical AE-based evaluation approach for demonstrating the fracture mechanism of asphalt concrete with different aggregate gradations.

## 1. Introduction

Asphalt concrete is a heterogeneous material consisting of multiple components, which can be divided into dense-graded asphalt concrete (AC), stone mastic asphalt (SMA), and open-graded friction course (OGFC), according to the varied compositions of gradation [1]. These three kinds of asphalt concrete have been extensively employed in pavement engineering in China. Different types of asphalt concrete possess different features to meet the actual engineering needs. The change in gradation can cause variations in the internal structure of asphalt concrete, showing different macro-performances [2]. Therefore, it is necessary to evaluate the damage characteristics of different types of asphalt concrete to improve service performance pertinently.

The mechanical performance of asphalt concrete is usually characterized by a resilient modulus, flow number, and indirect tensile strength [3,4]. Conventional approaches to evaluating the fracture characteristics of asphalt concrete, such as the single edge notch beam test, edge-cracked semi-circular bending test, and disc-shaped compact tension test [5,6,7], are somewhat limited, which are challenging to realize real-time monitoring throughout the damage process of asphalt concrete. However, the emergence of acoustic emission (AE) technology can make up for the above defects. AE is a phenomenon where the material releases energy rapidly through transient elastic waves when cracking and other activities that generate AE signals emerge [8]. During the failure process, the AE technique can obtain relevant parameters of the material to achieve real-time monitoring, and it has been applied diffusely in damage monitoring for rock [9], concrete [10], and pressure vessels [11].

However, limited work has been undertaken to validate the applicability of AE technology to characterize the damage mechanisms of asphalt concrete [12]. Arnold et al. [13] discussed the cracking characteristics of asphalt concrete, including different amounts of recycled asphalt shingles based on the AE technique. Jiao et al. [14,15,16] utilized AE parameters to evaluate the fracture modes of porous asphalt concrete. Qiu et al. [17] determined the AE waveform characteristics of asphalt concrete. McGovern et al. [18] and Sun et al. [19] discussed the fracture performance of asphalt concrete under various aging states with the aid of the AE technique. Li et al. [20] analyzed the influence of loading level on the failure process of asphalt concrete combined with AE tests and indirect tensile creep tests at low temperatures. Seo et al. [21] demonstrated the AE characterization during the fatigue failure process of asphalt concrete with cyclic loading tests. Velasquez et al. [22] evaluated the suitability of using taconite as aggregates in asphalt concrete with a series of experimental works with AE monitoring. Behnia et al. [23,24] investigated the effects of the cooling cycle and recycled asphalt pavement amounts on the cracking characteristics of asphalt concrete using the AE approach. Hill et al. [25] adopted AE parameters to evaluate the thermal cracking characteristics containing bio-modified asphalt concrete.

Previous research has only focused on one type of aggregate gradation to evaluate the AE characteristics of asphalt concrete during the failure process, where the effects of aggregate gradations on fracture characteristics of asphalt concrete with AE parameters have been neglected. The aggregate gradation has a significant impact on the mechanical properties of asphalt concrete [26,27]. Moreover, as a typical viscoelastic material, the damage characteristics of asphalt concrete are sensitively related to temperature. Therefore, the objective of this study was to investigate the AE characteristics of asphalt concrete with different aggregate gradations under different testing temperatures during the damage course.

In this study, specimens with three aggregate gradations containing AC, SMA, and OGFC were prepared in line with the Marshall compaction method. Indirect tensile tests (IDT) and AE tests were conducted simultaneously to explore the impact of aggregate gradations on the AE parameters, including energy, cumulative energy, count, and cumulative count during the damage process. Moreover, the temperature effect on asphalt concrete with different aggregate gradations was also demonstrated based on AE parameters.

## 2. Materials and Methods

### 2.1. Raw Materials

The aggregates and filler were both derived from limestone, and the specific indicators are listed in Table 1 following JTG E 42-2005 [28]. The 70 penetration-grade petroleum asphalt adopted in AC and SMA, SBS modified asphalt adopted in OGFC, and the specific indicators of these two kinds of asphalt are given in Table 2 according to JTG E20-2011 [29]. It can be seen that all indicators for aggregates and asphalt satisfied the specification requirements.

### 2.2. Aggregate Gradations and Specimens

**Figure 1:**
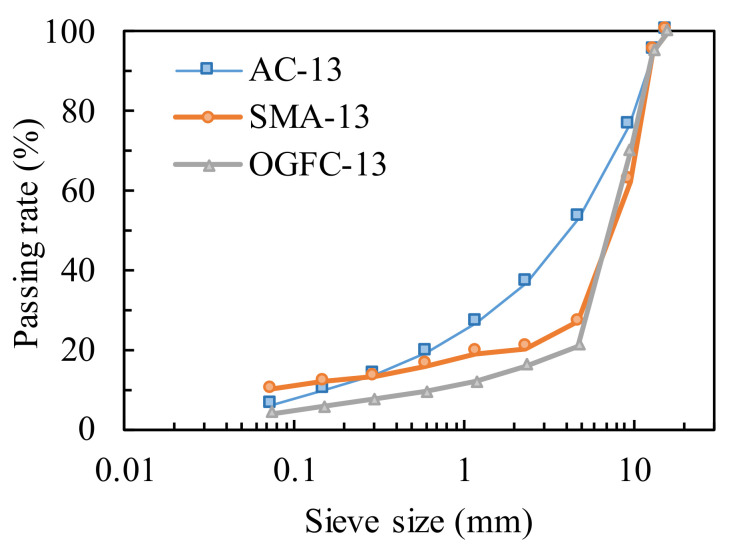
Selected gradation of AC, SMA, and OGFC.

Asphalt concrete with three types of aggregate gradations containing AC, SMA, and OGFC was prepared, and the maximum nominal size of each type of aggregate gradation was 13 mm. Figure 1 illustrates the corresponding grading curves selected according to JTG F40-2004 [30]. The optimal binder contents of AC, SMA, and OGFC, which were derived from the Marshall mix design method, were 5.0, 5.8, and 5.3, respectively. The specimens were double-sided compacted with 75 blows by the Marshall compaction method at the temperature of 150 °C. The corresponding diameter and height of specimens were (101.6 ± 0.2) mm and (63.5 ± 1.3) mm, respectively, which meets the requirement of ASTM D6931 for the size of the specimens in the indirect tensile test [31]. The basic volumetric parameters of specimens are illustrated in Table 3 following JTG E20-2011 [29].

### 2.3. Testing Procedure

**Figure 2:**
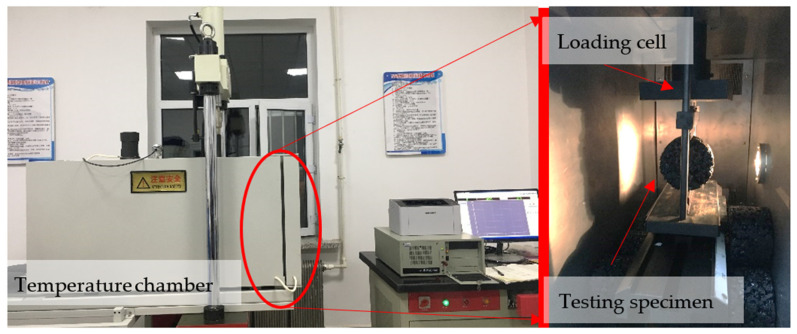
Universal testing machine. During the IDT process, asphalt concrete released energy from a local source and generated transient elastic waves, which is detected by the AE sensor and then amplified, processed, and recorded. By analyzing the recorded AE parameters, the damage mechanism of asphalt concrete could be characterized. The SAEU2S data acquisition system with six channels developed by Soundwel Technology Co. Ltd. (Beijing, China) was employed to conduct the test. The sampling accuracy of the acquisition card was ±1 dB, and the A/D conversion type was 16 bit, 10 MS/s. In order to filter out ambient noise during the acquisition process, the threshold value was set to 40 dB.

IDT was conducted with a universal testing machine (shown in Figure 2) produced by Sinotest Co. Ltd., Changchun, China. With the maximum loading level of 100 kN, this machine applied the load directly to the specimen through the electro-hydraulic servo actuator, and the corresponding load rate was 1 mm/min. The temperature chamber attached to the machine could realize temperature control within the range from −20 °C to 60 °C to ensure that the tests were conducted at the specific temperature, and relevant temperatures of IDT in this study were set to −10 °C and 25 °C, respectively. Specimens were placed in the incubator for 6 h to ensure the homogenous temperature distribution.

**Figure 3:**
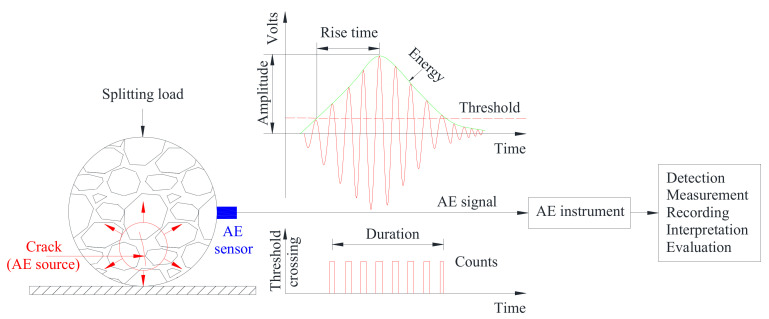
Schematic diagram of AE monitoring under indirect tensile load.

First, the SR150M ceramic AE sensor with frequency bands ranging from 60 kHz to 400 kHz and a resonant frequency of 150 kHz was immobilized on the side of the specimen by adhesive tape and coupled with grease. The pencil lead break was then carried out to ensure a favorable coupling condition between the sensor and specimen, after which the load was applied to the specimen and the AE parameters containing the energy and count during the fracture process were recorded by the AE instrument. The whole process is schematically presented in Figure 3.

### 2.4. AE Parameters

The fracture behavior of the three types of asphalt concrete was characterized by AE parameters containing energy, count, amplitude, rise time, and duration. It has been reported that all these parameters exhibited similar variations during the damage process of asphalt concrete [12]. In this study, the energy and count were selected to investigate the fracture characteristics of asphalt concrete with different aggregate gradations. AE energy is defined as the area enclosed by the oscillation curve of the AE signal and time axis, which can reflect the intensity of AE activity. AE count is the accumulation of oscillating pulse signals that exceed the threshold value, reflecting the intensity and frequency of AE activity.

## 3. Results and Discussions

### 3.1. Effect of Gradation on Fracture Characteristics of Asphalt Concrete

Three types of asphalt concrete with different gradations, including AC, SMA, and OGFC, were prepared. The nominal maximum size of an aggregate with different gradations was 13 mm, and the corresponding temperature of the IDT was set to 25 °C. AE parameters, including energy and count, were used to characterize the fracture behavior of specimens with different aggregate gradations. The fracture process of asphalt concrete can be split into several stages in terms of the variations in the AE parameters. The loads were uniformly transformed into load levels that intuitively characterized the correlation between mechanical and AE parameters. The value of load level can be calculated by Equation (1).

(1) $$\mathrm{Load}\mathrm{level}=\frac{F_{t}}{F_{m}}$$

where *F_t_* is the real-time load and *F_m_* is the maximum load.

#### 3.1.1. Dense-Graded Asphalt Concrete (AC)

**Figure 4:**
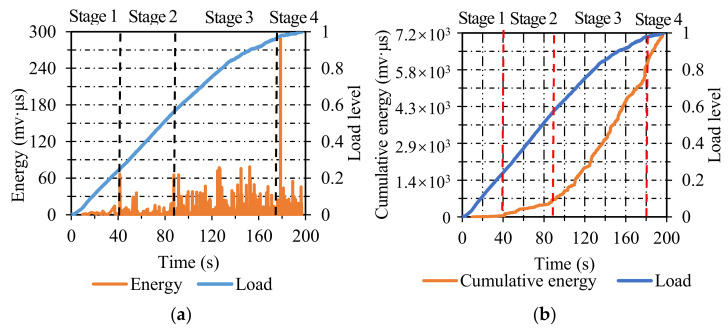
The energy and cumulative energy distribution of AC: (**a**) energy; (**b**) cumulative energy.

The AE energy distribution of AC specimens is presented in Figure 4a. The failure process can be divided into four stages. In stage one, only a small amount of energy appeared when the load level ranged from 0 to 0.2, which illustrated that only a small part of micro-cracks nucleated. In stage two, the corresponding load level increased from 0.2 to 0.55, and the energy was a little higher than the previous stage, but the energy value was still at a relatively low level, which illustrated that the micro-cracks expanded but did not form large cracks during this stage. In stage three, the energy was dense and maintained at a higher level when the load level increased from 0.55 to 0.95, which was related to the steady propagation of cracks. In the last stage, when the load level reached 0.95, an abrupt increase in energy value appeared, showing that the cracks expanded rapidly and led to the final fracture.

As seen in Figure 4b, the two curves exhibited a favorable correlation. The degradation of AC specimens can also be classified into four stages. In the first stage, the cumulative energy was maintained at approximately zero. In stage two, the cumulative energy increased gradually at a relatively low growth rate. In stage three, the growth rate of cumulative energy was higher than the previous one, and its load level lasted from 0.55 to 0.95. In stage four, the cumulative energy increased linearly in a short time until the final fracture of AC specimens.

**Figure 5:**
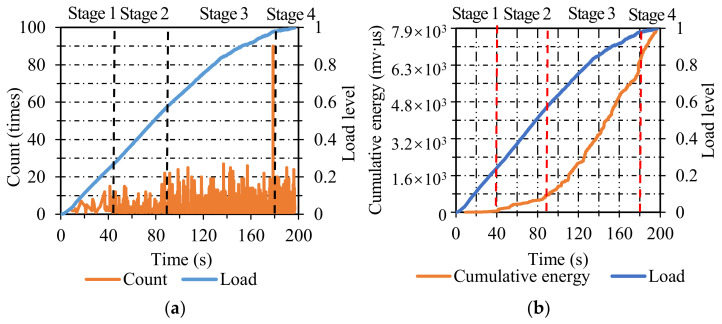
The count and cumulative count distribution of AC: (**a**) count; (**b**) cumulative count.

Figure 5a shows the count distribution of AC. The failure process can be split into four stages identically. In stage one, the AE count emerged sparsely at a low level, which was attributed to the nucleation of micro-cracks. In stage two, the AE count was kept at a slightly higher level than that in stage one when the load level ranged from 0.2 to 0.55, manifesting a small number of propagating cracks. In stage three, the AE count was maintained at a higher level than the previous stage when the load level increased from 0.55 to 0.95, which indicated the stable propagation of cracks. In stage four, a sudden increase in AE count appeared when the load level reached 0.95, which was associated with the final fracture of AC specimens.

Figure 5b represents the cumulative count distribution of AC. The two curves of load and cumulative count demonstrated favorable consistency. The damage course of AC specimens can be classified into four stages. In stage one, the value of the cumulative count was near zero when the load level ranged from 0 to 0.2. In stage two, the cumulative count rose gradually at a low-grade rate when the load level increased from 0.2 to 0.55. In stage three, it is evident that the cumulative count increased dramatically at a higher rate than the previous stage when the load level increased from 0.55 to 0.95 and lasted for a relatively long time. In stage four, the cumulative count ascended almost linearly in a short time, indicating the final fracture of AC specimens. Combining the variations in AE parameters (energy and count) and cumulative AE parameters (cumulative energy and cumulative count) in AC specimens, the energy and count were susceptible to identifying the final stage, and the cumulative energy and cumulative count were sensitive to recognizing the first three stages.

From a structural point of view, the traditional continuous dense-grade asphalt mixture is the suspend-dense structure, whose strength mainly derives from the cohesive force of the asphalt binder to aggregates. The asphalt concrete was subjected to a tensile force under indirect tensile load. In the initial stage, the asphalt binder and aggregate exhibited favorable cohesion and strong integrity, the cracking activities were not vigorous, and the phenomenon that the AE energy and count was at a relatively low level during the first three stages also confirmed this statement. When the load exceeded the tensile strength of specimens, final brittle fracture emerged, and the drastic increase in the AE energy and count in the final stage also authenticated this analysis.

#### 3.1.2. Stone Mastic Asphalt (SMA)

**Figure 6:**
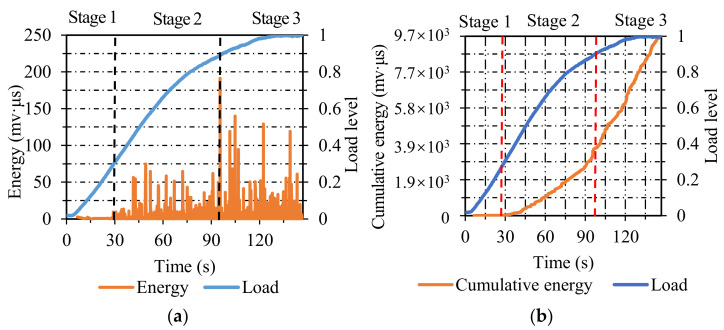
The energy and cumulative energy distribution of SMA: (**a**) energy; (**b**) cumulative energy.

Figure 6a illustrates the energy distribution of SMA. The damage course can be segmented into three stages. In stage one, the energy value was near zero before the load level reached 0.3, which indicated that only a small number of micro-cracks formed. In stage two, the energy emerged densely and was maintained at a relatively high level when the load level ranged from 0.3 to 0.9, demonstrating that the micro-cracks in SMA specimens expanded into macro-cracks steadily. In stage three, the energy value was maintained at a higher level than the previous stage when the load level increased from 0.9 to 1, which indicated that the cracks developed steadily and accumulated continuously until the final fracture.

Figure 6b shows the cumulative energy distribution. The fracture course of SMA specimens can be segmented into three stages. It can be seen that there was a limited cumulative energy in the initial stage before the load level reached 0.3. In stage two, the cumulative energy increased gradually when the load level increased from 0.3 to 0.9. In the last stage, the cumulative energy continued to increase at a high growth rate until the final fracture of SMA specimens.

**Figure 7:**
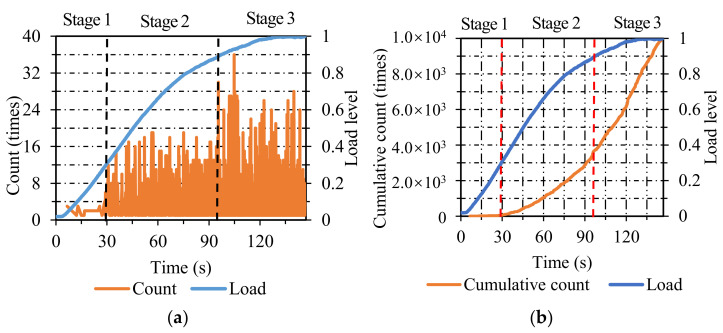
The count and cumulative count distribution of SMA: (**a**) count; (**b**) cumulative count.

As shown in Figure 7a, the damage process of SMA specimens can be split into three stages. In stage one, only a low count emerged before the load level reached 0.3, which was related to a small number of nucleated micro-cracks. In stage two, the AE count was at a relatively high level when the load level ranged from 0.3 to 0.9, indicating the stable expansion of micro-cracks to macro-cracks. In stage three, the AE count was maintained at a higher level than the previous stage when the load level ranged from 0.9 to 1, which was related to the steady and accumulative development of cracks until the final fracture.

The cumulative count distribution of SMA specimens is observed in Figure 7b. The failure process of SMA specimens can also be classified into three stages. In stage one, the cumulative count remained around zero before the load level reached 0.3. In stage two, the cumulative count increased gradually as the load level increased from 0.3 to 0.9. In the last stage, the cumulative count increased faster than the previous stage in terms of the curve slope. In terms of the comprehensive variations in AE parameters in SMA specimens, the energy and count exhibited satisfactory classification ability during the whole process, and it was vague for the cumulative energy and cumulative count to distinguish the demarcation point between stage two and stage three.

SMA specimens belong to the discontinuous gradation in grading types, and the gaps of the skeleton composed of coarse aggregates are filled with fine aggregates and asphalt binders in reasonable proportions, leading to the formation of the stable structure with an excellent packing effect. The internal friction and cohesion emerged to resist the displacement of asphalt concrete when the load was applied. The significant difference in AE characteristics between SMA and AC specimens lied in that the energy and count of SMA specimens were more uniform than those of AC specimens, and there was no drastic increase in AE parameters compared with AC specimens throughout the IDT process, which is attributed to the stable skeleton-dense structure.

#### 3.1.3. Open-Graded Friction Course (OGFC)

**Figure 8:**
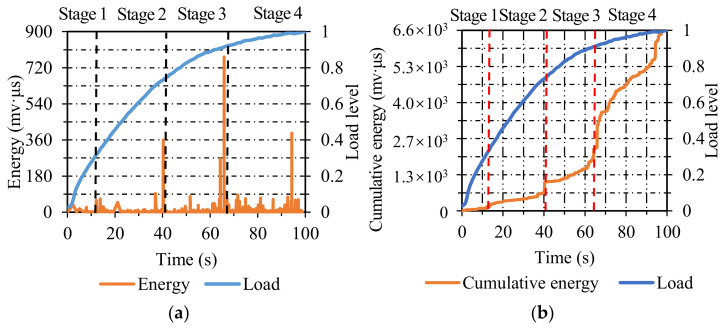
The energy and cumulative energy distribution of OGFC: (**a**) energy; (**b**) cumulative energy.

Figure 8a shows the energy distribution of OGFC specimens. The damage process of OGFC specimens can be classified into four stages. In stage one, only a small amount of energy emerged before the load level reached 0.3, representing the generation of micro-cracks. In stage two, the energy was at a higher level than the former stage as the load level ranged from 0.3 to 0.75, which can be considered as the propagation of micro-cracks. There was a sudden rise when the load level reached 0.75, 0.9, and 0.98, manifesting stress concentration resulted in large cracks in OGFC specimens. In stage three, the energy emerged densely but was still at a low level when the load level increased from 0.75 to 0.9, which illustrated stable crack growth. In stage four, the energy exhibited a denser distribution than the previous stage, which contributed to the final fracture of OGFC specimens.

As seen in Figure 8b, the damage process can be divided into four stages. In stage one, it is apparent that the cumulative energy was at a low level. In stage two, the cumulative energy increased gradually as the load level increased from 0.3 to 0.75. In stage three, the cumulative energy ascended steadily at a higher growth rate than that of stage two. In the last stage, the cumulative energy exhibited a more precipitous ascending trend than that of the previous stage until the final fracture of OGFC specimens.

**Figure 9:**
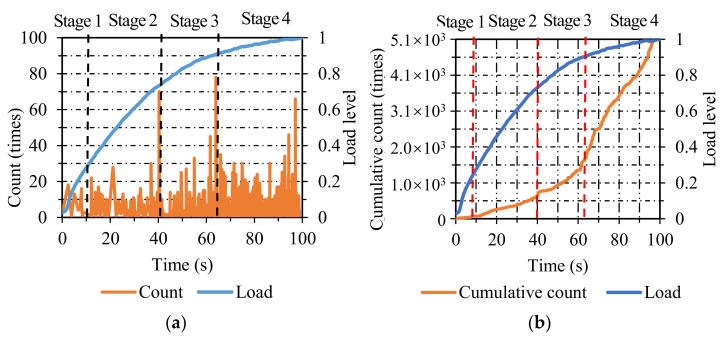
The count and cumulative count distribution of OGFC: (**a**) count; (**b**) cumulative count.

Figure 9a represents the count distribution of OGFC specimens. The failure course of OGFC specimens can be classified into four stages. In stage one, only a low count appeared when the load level ranged from 0 to 0.3, which indicated the generation of micro-cracks. In stage two, the AE count remained at a relatively high level as the load level ranged from 0.3 to 0.75, manifesting the propagation of cracks. In stage three, the energy was maintained at a higher level than the previous stage when the load level increased from 0.75 to 0.9, which can be considered as the expansion of cracks. In stage four, the energy distributed densely after the load level reached 0.9 and gradually resulted in the final fracture of OGFC specimens.

As shown in Figure 9b, the damage process of OGFC specimens can be split into four stages. In stage one, the cumulative count was maintained at a low-grade level as the load level increased from 0 to 0.3. In stage two, the cumulative count increased gradually as the load level increased from 0.3 to 0.75. In stage three, the cumulative count ascended continuously with a higher rate of ascent compared with stage two when the load level increased from 0.75 to 0.9. In stage four, the cumulative count exhibited an almost linear growth after the load level reached 0.9, which contributed to the final destruction of OGFC specimens. Among the overall variations in AE parameters in OGFC specimens, the cumulative energy and cumulative count exhibited favorable identification abilities throughout the failure process, and it was fuzzy for energy and count to distinguish the demarcation point between stage one and stage two.

OGFC specimens belong to the continuous open gradation in gradation type, and it is a skeleton-void structure with large air voids, where the structure is composed of a large number of course aggregates and a small number of fine aggregates. The voids between the skeleton cannot be adequately filled by fine aggregates, which leads to the inability to form a strong interlocking effect between the particles. The strength of OGFC specimens mainly relies on the cohesive force between the binder and aggregates. The air voids of OGFC specimens are relatively large, and the contact state between particles is stone-to-stone, making the OGFC specimens prone to stress concentration in the IDT process. The cracks occurred and released a considerable quantity of AE energy when the stress concentration accumulated to a critical extent. Thus, the energy and count mutation points of OGFC specimens were intermittently distributed throughout the IDT process.

The combined analysis of AE parameters and cumulative AE parameters enables the accurate classification of the fracture process in asphalt concrete. These three types of asphalt concrete exhibited different fracture characteristics during the failure process owing to their dissimilar skeletal structures. The internal damage of AC specimens developed slowly in the early loading stages but accumulated sharply in the final stage until they were completely destroyed. The SMA specimens displayed a steady development of internal damage under loading until the final fracture. The OGFC specimens were accompanied by localized damage throughout the failure process due to stress concentration.

### 3.2. Effect of Testing Temperature on Fracture Characteristics of Asphalt Concrete

Energy and cumulative energy were employed to assess the influence of testing temperature on the damage characteristics of specimens. The IDT and AE tests were conducted simultaneously at the temperatures of −10 °C and 25 °C, respectively. Time data were converted into time levels to characterize the variation in AE parameters visually with temperature. The value of the time level can be calculated by Equation (2).

(2) $$\mathrm{Time}\mathrm{level}=\frac{t}{t_{o}}$$

where *t* is the loading time and *t_o_* is the overall loading time.

#### 3.2.1. Dense-Graded Asphalt Concrete (AC)

**Figure 10:**
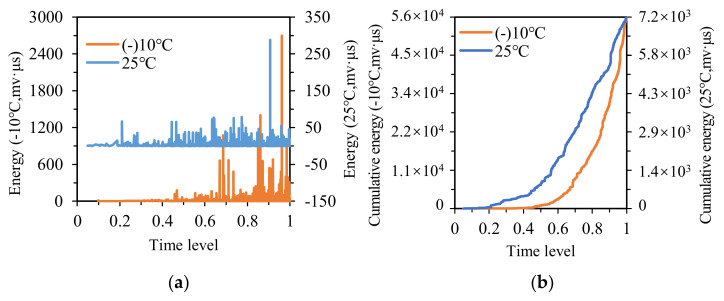
The energy and cumulative energy distribution of AC at −10 °C and 25 °C: (**a**) energy; (**b**) cumulative energy.

Figure 10a illustrates the energy distribution of AC specimens. The energy value at −10 °C was much higher than that at 25 °C, which was ascribed to the viscoelastic attribute of asphalt concrete. Compared with high temperatures, the asphalt concrete was more brittle and exhibited more intense AE activities at low temperatures. Besides, the variations in energy versus time level were significantly influenced by temperature. The energy of AC specimens began to emerge densely approximately at the time levels of 0.45 and 0.2 at −10 °C and 25 °C, respectively.

As seen in Figure 10b, the initial rising point of cumulative energy at −10 °C lagged behind that at 25 °C, the cumulative energy began to rise at the time level of 0.5 at −10 °C, while it started to rise at the time level of 0.2 at 25 °C. The variation in energy and cumulative energy versus time level at different temperatures displayed favorable consistency.

As mentioned above, the AC specimens possess the suspend-dense structure, whose strength mainly derives from the cohesive force of asphalt binder to aggregates. The change in temperature leads to the variety of cohesion between binders and aggregates, which affects the mechanical performance of specimens. Thus, the change in temperature significantly affects the AE parameters of asphalt concrete during its failure process.

#### 3.2.2. Stone Mastic Asphalt (SMA)

**Figure 11:**
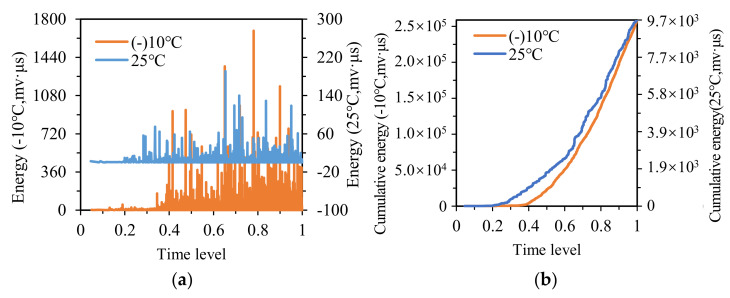
The energy and cumulative energy distribution of SMA at −10 °C and 25 °C: (**a**) energy; (**b**) cumulative energy.

Figure 11a shows the energy distribution. Similarly, the energy value at −10 °C was much higher than that at 25 °C. The energy of SMA specimens began to emerge densely at the time levels of 0.35 and 0.25 at −10 °C and 25 °C, respectively, and the variations in energy versus time level exhibited favorable consistency at these two temperatures.

The cumulative energy distribution is presented in Figure 11b, the two curves at −10 °C and 25 °C showed a similar tendency, and the initial rising point of cumulative energy at −10 °C was slightly later than that at 25 °C. Besides, the area surrounded by the two curves can be employed to characterize the temperature stability of specimens. The smaller the area, the better the temperature stability of specimens. Conversely, the larger the area, the worse the temperature stability of specimens. As seen in Figure 10b and Figure 11b, the area enclosed by the two curves of SMA specimens was significantly smaller than that of AC specimens, indicating that the SMA specimens possess better temperature stability than AC specimens.

The strength of SMA specimens mainly derives from internal friction and cohesion. The change in temperature affects the cohesion of SMA specimens to a certain extent, but SMA specimens rely more on the internal friction of the skeleton-embedded structure to bear the load. Thus, the variation in energy and cumulative energy versus time is less affected by temperature.

#### 3.2.3. Open-Graded Friction Course (OGFC)

**Figure 12:**
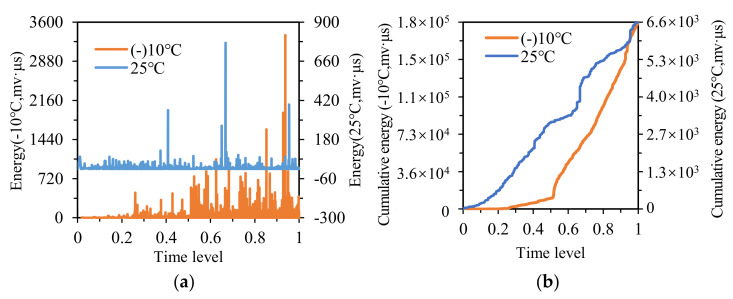
The energy and cumulative energy distribution of OGFC at −10 °C and 25 °C: (**a**) energy; (**b**) cumulative energy.

The energy distribution of OGFC specimens is illustrated in Figure 12a, and the energy value at −10 °C was much higher than that at 25 °C. The energy of OGFC specimens began to emerge densely approximately at the time level of 0.25 at −10 °C. However, at 25 °C, the energy emerged densely at the initial stage of IDT and lasted until the final fracture, and the energy value at 25 °C occurred continuously earlier than that at −10 °C.

Figure 12b shows the cumulative energy distribution of OGFC specimens at −10 °C and 25 °C, where the initial rising point of cumulative energy at −10 °C lagged significantly behind that at 25 °C, the curve at −10 °C started to rise at the time level of 0.3, while it started to rise at the initial time level at 25 °C. As seen in Figure 10b, Figure 11b and Figure 12b, the area enclosed by two cumulative energy curves at −10 °C and 25 °C of OGFC specimens was larger than that of SMA specimens and close to that of AC specimens, indicating that the SMA specimens possess the best temperature stability among the three types of asphalt concrete.

The strength of OGFC specimens mainly relies on the cohesive force between binders and aggregates. The change in temperature leads to the change in cohesion between the binder and aggregates, causing the fluctuation in mechanical properties. Thus, the temperature shows a significant impact on the AE parameters of OGFC specimens during the IDT process.

The AE technique provides a new method to estimate the temperature stability of asphalt concrete. Combining the variations in AE parameters of these three types of asphalt concrete at different temperatures, it can be seen that the AE activities at −10 °C were more intense and the internal damage of asphalt concrete developed later compared to 25 °C. In addition, the SMA specimens exhibited the best temperature stability based on the AE parameter analysis. Compared with the traditional mechanical parameters, the AE parameters were more sensitive to the internal damage of asphalt concrete, which could help us understand the fracture mechanisms of asphalt concrete comprehensively.

## 4. Conclusions

In this study, the fracture characteristics of asphalt concrete with three aggregate gradations, including AC, SMA, and OGFC, under IDT using the AE technique were demonstrated, and the temperature effects of asphalt concrete characterized by AE parameters were also discussed. The most relevant conclusions are:

1. Asphalt concrete with different aggregate gradations exhibited different fracture characteristics based on AE monitoring, and the comprehensive analysis of AE parameters and cumulative AE parameters can realize accurate identification of the damage process of asphalt concrete.
2. The damage fracture of AC, SMA, and OGFC specimens can be divided into four, three, and four stages due to AE parameters, respectively. As for AC specimens, the fracture characteristics were manifested by the sudden fracture due to insufficient cohesion under critical load. The fracture process of SMA specimens was characterized by the stable propagation of cracks owing to the stable skeleton-dense structure. Due to the high voids content, the fracture characteristics of OGFC specimens were represented as the local failure caused by the stress concentration related to the skeleton-void structure throughout the IDT process.
3. The energy and cumulative energy of AC, SMA, and OGFC specimens varied with temperature, and the SMA specimens displayed the best overall performance among these three kinds of asphalt concrete in terms of the distribution of AE parameters at different temperatures.

## Figures and Tables

**Table 1:** Properties of aggregates and filler.

Items	Coarse Aggregate	Fine Aggregate	Filler	Technical Criterion	Method
Crushing value (%)	14.4	-	-	≤26	T0316
Los Angeles abrasion loss (%)	18.6	-	-	≤28	T0317
Apparent specific gravity (%)	3.21	2.95	2.73	≥2.5	T0304
Sand equivalent (%)	-	82	-	≥60	T0334
Plasticity index (%)	--	-	2.8	≤4.0	T0354

**Table 2:** Properties of matrix asphalt (MA) and SBS-modified asphalt (SBS-MA).

Items	MA	Technical Criterion	SBS-MA	Technical Criterion	Method
Penetration (25 °C, 0.1 mm)	72	60–80	64	60–80	T0604
Softening point (°C)	47.5	≥45	64.2	≥55	T0606
Ductility (15 °C, cm)	145	≥45	-	-	T0605
Ductility (5 °C, cm)	-	-	34.5	≥30	T0605
Density (g/cm^3^)	1.036	-	1.065	-	T0603
Flashing point (°C)	275	≥260	264	≥230	T0611

**Table 3:** Volumetric parameters of specimens.

Items	AC-13	Technical Criterion	SMA-13	Technical Criterion	OGFC-13	Technical Criterion	Method
Air voids (%)	3.9	3–5	3.3	3–4	20.8	18–25	T0705
Voids in mineral aggregates (%)	13.56	≥13	19.54	≥17	31.73	-	T0705
Voids filled with asphalt (%)	71.23	65–75	83.11	75–85	27.83	-	T0705
Theoretical maximum specific density	2.522	-	2.551	-	2.602	-	T0705
Bulk specific gravity	2.424	-	2.467	-	2.061	-	T0705

## Data Availability

The data presented in this study are available on request from the corresponding author.
